# Generation and characterization of a temperature-sensitive mutant allele of the second largest subunit of RNA polymerase I in *Schizosaccharomyces pombe*

**DOI:** 10.17912/micropub.biology.000586

**Published:** 2022-06-10

**Authors:** Kazuki Ishida, Katsunori Tanaka, Kei Kawakami

**Affiliations:** 1 Department of Bioscience, Graduate School of Science and Technology, Kwansei Gakuin University, Sanda, Hyogo, Japan; 2 Department of Biosciences, School of Biological and Environmental Sciences, Kwansei Gakuin University, Sanda, Hyogo, Japan

## Abstract

RNA polymerase I (Pol I) is a highly conserved complex that catalyzes the transcription of rRNA precursors in the nucleolus. In this study, we isolated a temperature-sensitive (ts) allele of Rpa2, the second largest subunit of Pol I in the fission yeast *Schizosaccharomyces pombe* . We found that *rpa2* ^ts^ cells were severely defective in growth at temperatures above 32 °C. We also found that *rpa2* ^ts^ cells showed aberrant chromosome segregation and an abnormal ring-like nuclear structure at the restrictive temperature. These findings suggest that Rpa2 is essential for faithful nuclear division and nuclear structural organization in *S. pombe* .

**Figure 1. Generation and characterization of a temperature-sensitive mutant allele of the second largest subunit of RNA polymerase I:**
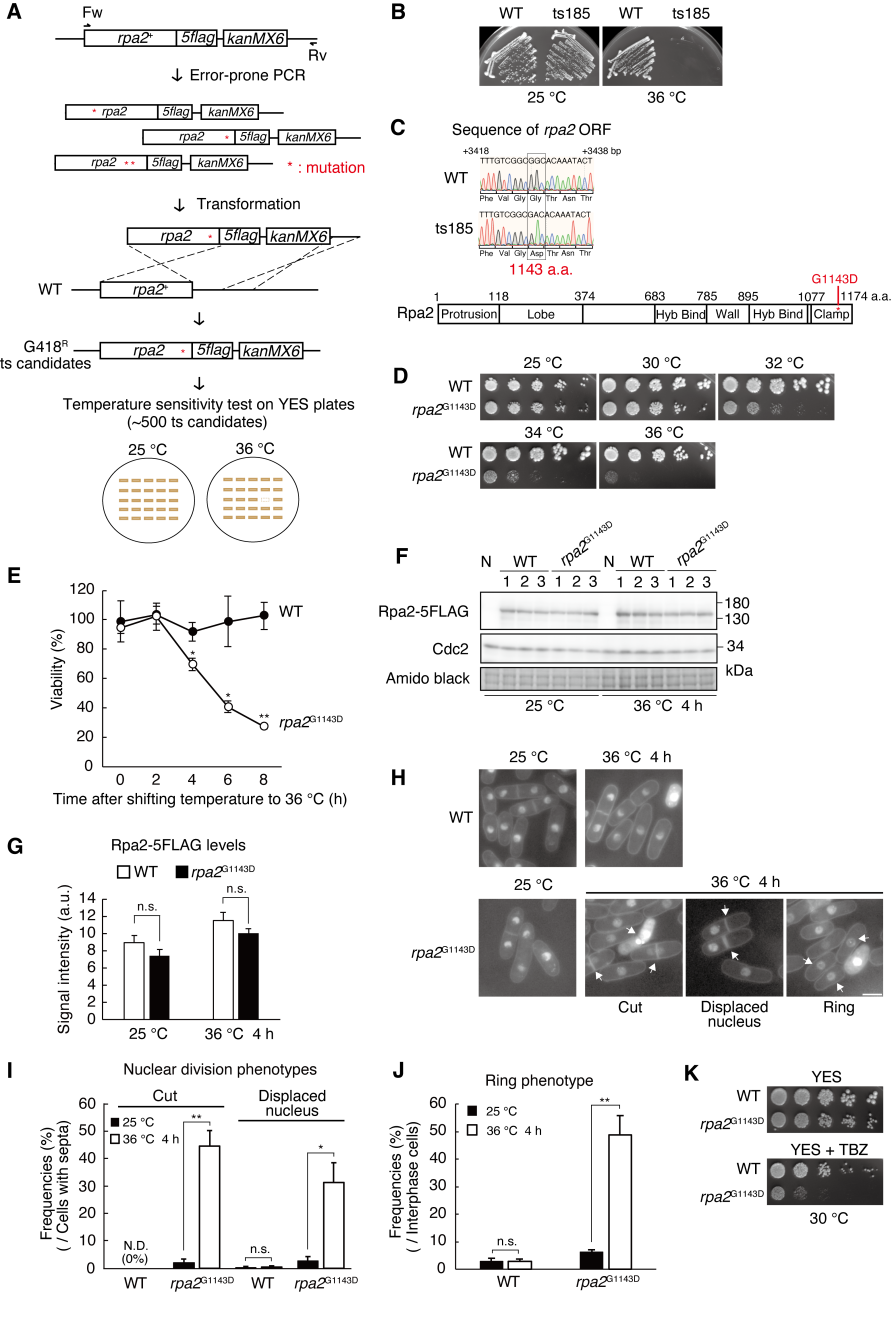
(A) Strategy to generate the temperature-sensitive allele of *rpa2* using error-prone PCR. Wild-type cells were transformed with randomly mutated *rpa2-5flag* :: *kanMX6* DNA fragments. Temperature-sensitive (ts) transformants were isolated from G418 resistant (G418 ^R^ ) candidates. Refer to the Methods section for details. (B) Growth test of ts185. Wild-type ( *rpa2-5flag* :: *kanMX6* ) and ts185 cells were streaked onto YES plates and incubated at 25 or 36 °C for 3 d. (C) Sequencing analysis of *rpa2* ORF in ts185. Glycine at amino acid position 1143 in Rpa2 was substituted with aspartic acid (G1143D) in ts185 (upper panel). Domain structure of Rpa2 (lower panel). See the ref. (Heiss et al. 2021) for a complete description of the domains that are not presented here. (D) Growth test of *rpa2* ^G1143D^ at various temperatures. Five-fold serial dilutions of wild-type ( *rpa2-5flag* :: *kanMX6* ) and *rpa2* ^G1143D^ ( *rpa2* ^G1143D^ *-5flag* :: *kanMX6* ) cells were spotted onto YES plates and incubated for 3 d at 25, 30, 32, 34, or 36 °C. (E) Measurement of cell viability after shifting to restrictive temperature. Approximately 250–500 cells of wild-type ( *rpa2-5flag* :: *kanMX6* ) and *rpa2* ^G1143D^ ( *rpa2* ^G1143D^ *-5flag* :: *kanMX6* ) were collected and spread onto YES plates. After incubation for 4 d at 25 °C, the number of colonies was counted. Error bars show the standard deviation from three independent cultures. Statistical significance was determined by Student’s t-test. (* *p* < 0.05, ** *p* < 0.01). (F) Western blotting analysis of Rpa2-5FLAG. Total proteins were extracted from wild-type ( *rpa2-5flag* :: *kanMX6* ) and *rpa2* ^G1143D^ ( *rpa2* ^G1143D^ *-5flag* :: *kanMX6* ) cells at 25 °C or after 4 h incubation at 36 °C. Total proteins were prepared from three independent cultures (lane 1–3). Samples were analyzed with anti-FLAG IgG. Anti-Cdc2 IgG blotting and amido black staining of the membrane were served as loading controls. N represents the untagged negative control. (G) The quantification of Rpa2-5FLAG protein levels in (F). The signal intensities corresponding to Rpa2-5FLAG bands were quantified by the Image J software. The intensity of each band was normalized by the signal intensity of amido black staining. The vertical axis shows the signal intensity in arbitrary units. Error bars show the standard deviation from three samples prepared from independent cultures. Statistical significance was determined by Student’s t-test. (n. s. = not significant). (H) Phenotypic observation of wild-type ( *rpa2-5flag* :: *kanMX6* ) and *rpa2* ^G1143D^ ( *rpa2* ^G1143D^ *-5flag* :: *kanMX6* ) cells incubated at 25 or 36 °C for 4 h under the microscope. Nuclear chromatin regions were stained with DAPI. The white bar represents 5 μm. White arrows in each panel show cells with typical phenotypes, as shown in the images. (I) The frequencies of the nuclear division phenotypes shown in (H). To calculate the frequencies of cells with *cut* or displaced nuclei, the number of cells with septa was calculated as the population (n ≥ 100). Error bars show the standard deviation from three independent cultures. Statistical significance was determined by Student’s t-test. (* *p* < 0.05, ** *p* < 0.01, N. D. = not detected, n. s. = not significant). (J) The frequencies of the ring phenotypes shown in (H). To calculate the frequencies of cells with a ring phenotype, the number of cells at the interphase was calculated as the population (n > 150). Error bars show standard deviation from three independent cultures. Statistical significance was determined by Student’s t-test. (** *p* < 0.01, n. s. = not significant). (K) Assessment of TBZ sensitivity. Five-fold serial dilutions of wild-type ( *rpa2-5flag* :: *kanMX6* ) and *rpa2* ^G1143D^ ( *rpa2* ^G1143D^ *-5flag* :: *kanMX6* ) cells were spotted onto YES plate or YES plate containing 15 μg/mL TBZ and incubated for 4 d at 30 °C.

## Description

In all eukaryotes, Pol I has long been known to be a conserved complex that catalyzes rRNA transcription in the nucleolus. Recently, genes encoding several Pol I subunits have been identified to be responsible for Treacher Collins syndrome, and their importance in genetic diseases has been highlighted (Dauwerse et al. 2011). However, genetic analyses of Pol I have not been performed sufficiently. In the yeast species *Schizosaccharomyces pombe* and *Saccharomyces cerevisiae* , Pol I consists of 14 subunits, including the two largest subunits that form the polymerase active center (Fernández-Tornero et al. 2013; Heiss et al. 2021). The Pol I active center is regulated by two parts: the central cleft that binds the DNA template and the clamp that opens and closes the cleft (Fernández-Tornero et al. 2013; Heiss et al. 2021). Nuc1 is the largest subunit of *S. pombe* and RPA190 in *S. cerevisiae* . The second largest subunit is known as Rpa2 in *S. pombe* and RPA135 in *S. cerevisiae* . In *S. cerevisiae* , conditional mutants have been isolated and analyzed for RPA190 and RPA135 (Nogi et al. 1991; Wittekind et al. 1988) *.* Although *S. pombe* is also an excellent model organism as *S. cerevisiae* , of the two largest subunits, conditional mutants of only Nuc1 have been established and analyzed in case of *S. pombe* (Hirano et al. 1986; Hirano et al. 1989). Therefore, we generated a temperature-sensitive mutant allele of *rpa2* to augment the genetic analysis of Pol I in *S. pombe* .

We used the error-prone PCR method to introduce random mutations into the ORF of *rpa2* . In this method, the entire *rpa2* ORF with *5flag* :: *kanMX6* C-terminal tagging module was amplified using PCR with an excessive number of amplification cycles in the presence of high concentrations of dNTPs **(Figure 1A)** (refer to the Methods section for details). Under these conditions, DNA polymerase tends to generate errors at a relatively high frequency and can efficiently produce a pool of randomly mutated *rpa2-5flag* :: *kanMX6* fragments **(Figure 1A)** . The wild-type strain was then transformed with mutated *rpa2-5flag* :: *kanMX6* fragments. We screened for mutants that could grow at 25 °C but not at 36 °C **(Figure 1A)** . We screened ~500 G418 ^R^ transformants and succeeded in isolating a mutant, ts185, exhibiting the desired phenotype **(Figure 1A and B)** . After confirming that the *rpa2* allele was linked to the temperature-sensitive (ts) phenotype by backcrossing, sequencing was performed to identify the mutation site. From these results, we identified a G-to-A base substitution within the ORF of *rpa2* . This single-base substitution caused an alteration of glycine at position 1143 to aspartic acid (G1143D) in the C-terminal region **(Figure 1C)** . Mutations other than G1143D were not observed in the ORF of *rpa2* . We named this novel ts allele “ *rpa2* ^G1143D^ *.”* From previous crystallographic analysis, the C-terminal region of Rpa2, where G1143 is located, was shown to be located at the clamp domain of the Pol I active center **(Figure 1C)** (Heiss et al. 2021).

To analyze the *rpa2* ^G1143D^ phenotype in detail, we performed spot assays at various temperatures. We found that *rpa2* ^G1143D^ almost grew similarly to the wild-type strain at 25 °C and 30 °C, but showed severe growth defects even at 32 °C, and it was nearly impossible to form colonies at 34 °C and 36 °C **(Figure 1D)** . From these results, we concluded that temperatures below 30 °C were permissive, those at 32 °C was semi-permissive, and those over 34 °C were restrictive for *rpa2* ^G1143D^ cells. It should be noted that the *rpa2* ^G1143D^ cells formed slightly smaller colonies than the wild-type, even at permissive temperatures **(Figure 1D)** . This result suggested that the G1143D mutation can partially affect Rpa2 function even at permissive temperatures. Next, to determine whether *rpa2* ^G1143D^ cells stopped proliferating or died at the restrictive temperature, we measured cell viability **(Figure 1E)** . After the incubation temperature was increased from 25 °C to 36 °C, the cell viability of *rpa2* ^G1143D^ began to reduce after 4 h of incubation and dropped to approximately 25 % in 8 h **(Figure 1E)** . This result suggested that restrictive temperatures are lethal to *rpa2* ^G1143D^ cells. To analyze whether the G1143D mutation affects Rpa2 protein levels, we performed western blotting analysis against total proteins extracted from wild-type and *rpa2* ^G1143D^ cells at 25 °C and after 4 h incubation at 36 °C. There was no significant change in the amount of Rpa2-5FLAG protein at either the permissive or restrictive temperatures **(Figure 1F and G)** , suggesting that the G1143D mutation causes the loss of Rpa2 functions without affecting protein stability at the restrictive temperature.

To understand how *rpa2* ^G1143D^ cells lose viability at the restrictive temperature, we observed the cytological phenotypes of *rpa2* ^G1143D^ cells by staining their nuclei with DAPI. Various abnormalities associated with nuclear division were observed in the *rpa2* ^G1143D^ cells after 4 h of incubation at 36 °C **(Figure 1H)** . Nuclear division phenotypes were classified into two categories: the first was “ *cut* ” phenotype in which the septum cut across the undivided chromosomes and the second was “displaced nucleus” phenotype in which the nucleus was present in only one of the two cells separated by a septum **(Figure 1H)** . We found that the frequencies of each phenotype were less than 6 % at the permissive temperature **(Figure 1I)** . After 4 h of incubation at 36 °C, the frequencies of *cut* and displaced nucleus phenotypes increased to approximately 45 % and 31 %, respectively **(Figure 1I)** . In wild-type cells, these two phenotypes were rarely observed (~1 %) **(Figure 1I)** . Because the viability of *rpa2* ^G1143D^ cells reduced after 4 h of incubation at the restrictive temperature, it was strongly suggested that chromosome segregation errors cause cell death. Consistent with chromosome segregation defects, *rpa2* ^G1143D^ cells were sensitive to thiabendazole (TBZ), a microtubule-depolymerizing drug **(Figure 1K)** , suggesting that Rpa2 is essential for faithful nuclear division. Next, we focused on the shape of the interphase nuclei. In *rpa2* ^G1143D^ cells, the interphase nuclear chromatin region was hemispherical at the permissive temperature, as observed in the wild-type cells **(Figure 1H)** . However, it was altered into hollow, ring-like structures in approximately 49 % of interphase *rpa2* ^G1143D^ cells (called “ring phenotype”) at the restrictive temperature (Hirano et al. 1989) **(Figure 1H and J)** , suggesting that Rpa2 function is essential for the normal nuclear structural organization.

Previously, it had been reported that double mutants of *top1* and *top2* that encode DNA topoisomerase I and II, respectively, showed ring phenotypes such as *rpa2* ^G1143D^ (Uemura and Yanagida 1984). Interestingly, *top2* single mutant showed the *cut* phenotype (Uemura and Yanagida 1984). Based on the phenotypic similarity between *rpa2* ^G1143D^ and DNA topoisomerase mutants, it is possible that Rpa2 is involved in DNA topoisomerase function. Previous studies have shown that the temperature-sensitive mutant of *nuc1* has a collapsed nucleolus, showing a ring phenotype similar to *rpa2* ^G1143D^ (Hirano et al. 1986; Hirano et al. 1989). Thus, the two largest subunits of Pol I have essential functions in the normal nuclear structural organization. Another study showed that Nuc1 plays an important role in accumulating a condensin subunit, Cut14, at rDNA repeats during mitosis (Nakazawa et al. 2008). A temperature-sensitive mutant of *cut14* failed to separate rDNAs into daughter cells and showed the *cut* phenotype (Nakazawa et al. 2008; Samejima et al. 1993). Thus, Rpa2 may also play an important role in condensin accumulation at rDNA repeats for faithful chromosome segregation. Further studies are required to elucidate the detailed function of Rpa2 during mitosis.

In this study, we succeeded in generating the ts allele of *rpa2* **(Figure 1A-D)** and showed that Rpa2 is essential for nuclear division and nuclear structural organization **(Figure 1H-K)** . To our knowledge, this study is the first to generate and characterize the ts allele of *rpa2* in *S. pombe* . Recently, POLR1B, the human counterpart of Rpa2, was identified as the gene responsible for Treacher Collins syndrome (Sanchez et al. 2020). Because the features of cell division and chromatin structures in *S. pombe* are thought to be more similar to those of vertebrates than *S. cerevisiae* (Wood et al. 2002), studies using *rpa2* ^G1143D^ may provide fundamental insights into the above-mentioned important issues of biological interest and human diseases.

## Methods

*Schizosaccharomyces pombe* media and genetic procedures

The media and genetic procedures used in this study have been described previously (Moreno et al. 1991). C-terminal 5FLAG epitope tagging for Rpa2 was performed as previously described (Krawchuk and Wahls 1999).

Error-prone PCR

A temperature-sensitive allele of *rpa2* was generated as previously described (Kato et al. 2008; Santosa et al. 2013). Briefly, genomic DNA derived from the wild-type *rpa2-5flag* :: *kanMX6* strain was used as a template to amplify the entire *rpa2-5flag* :: *kanMX6* sequence using Ex Taq (Takara Bio, Shiga, Japan). To achieve efficient mutagenesis, the standard protocol was modified as follows: (1) the number of amplification cycles was set to 40, and (2) the final concentration of dNTPs in the reaction was doubled to 0.4 mM each. The primers used for amplifying the *rpa2-5flag* :: *kanMX6* fragment are listed below in the Reagents section. The lithium acetate method was used for this transformation.

Spot assay

Cells were grown to the stationary phase in YES medium at 25 °C, and five-fold serial dilutions were prepared and spotted onto YES plates. The cells were incubated for 3 d at 25, 30, 32, 34, or 36 °C before imaging. For drug sensitivity analysis, the cells were spotted onto YES plates containing 15 μg/mL TBZ (MP Biomedicals, Irvine, CA, USA) and incubated for 4 d at 30 °C.

Measurement of cell viability

Cells were grown to the log phase at 25 °C in YES medium. After adjusting their density to 1 × 10 ^6^ cells/mL, the cells were re-cultured and incubated at 36 °C. A total of 250–500 cells were collected every 2 h and spread onto YES plates. After 4 d of incubation at 25 °C, the number of colony-forming cells was counted.

Microscopic analysis

Exponentially growing cells in YES medium were fixed with 2.5 % glutaraldehyde (FUJIFILM Wako, Osaka, Japan) for 30 min on ice. The cell pellet was washed three times with ice-cold phosphate-buffered saline. The cell suspensions were mixed with equal volumes of 50 μg/mL DAPI (DOJINDO, Kumamoto, Japan). Images of one section with the largest nuclear diameter were captured using a fluorescence microscope BX51 (OLYMPUS, Tokyo, Japan).

Protein extraction, western blotting, signal intensity quantification

Cells cultured in YES medium at 25 or 36 °C were harvested for 7 x 10 ^7^ cells. Cell pellets were re-suspended in 150 μL of 2 × SDS sample buffer together with glass beads. Cells were lysed with MULTI BEADS SHOCKER (YASUI KIKAI, Osaka, Japan). To denature proteins, samples were boiled at 95 °C for 5 min. For western blotting, HRP-conjugated anti-FLAG IgG (M2) (Sigma-Aldrich, St. Louis, MO, USA) was used to detect Rpa2-5FLAG. Anti-Cdc2 IgG (B-6) (Santa Cruz, Dallas, TX, USA) and HRP-conjugated anti-rabbit IgG (Promega, Madison, WI, USA) were used as primary or secondary antibodies, respectively, to detect Cdc2. Image J software (https://imagej.nih.gov/ij/) was used to quantify the signal intensity of Rpa2-5FLAG. After background subtraction, Rpa2-5FLAG signal intensities were normalized by the signal ratio of amido black staining.

## Reagents

All the strains used in this study and their genotypes are listed below.

**Table:** 

**Strain name**	**Genotype**	**Origin**
PR109	* h ^-^ leu1-32 ura4-D18 *	P. Russell Lab
Bio9308	* h ^-^ leu1-32 ura4-D18 rpa2-5flag* :: *kanMX6*	This study
Bio9088	* h ^-^ leu1-32 ura4-D18 rpa2* ^G1143D^ *-5flag* :: *kanMX6*	This study

The primers used in error-prone PCR are listed below.

**Table:** 

**Primer name**	**Sequence (5′-3′)**
KT3046 *rpa2* error-prone PCR Fw	ATGTCATTCCAAACGTTAGAGC
KT2961 *rpa2* error-prone PCR Rv	CATACGAATGTGTGCTCACG
